# Non-linear Relationship between BOLD Activation and Amplitude of Beta Oscillations in the Supplementary Motor Area during Rhythmic Finger Tapping and Internal Timing

**DOI:** 10.3389/fnhum.2017.00582

**Published:** 2017-11-30

**Authors:** Florian Gompf, Anja Pflug, Helmut Laufs, Christian A. Kell

**Affiliations:** ^1^Cognitive Neuroscience Group, Department of Neurology, Brain Imaging Center, Goethe University Frankfurt, Frankfurt am Main, Germany; ^2^Department of Neurology, University Hospital Schleswig-Holstein, Campus Kiel, Christian-Albrechts- Universität zu Kiel, Kiel, Germany

**Keywords:** EEG-fMRI, predictive timing, internal time, supplementary motor area (SMA), premotor cortex

## Abstract

Functional imaging studies using BOLD contrasts have consistently reported activation of the supplementary motor area (SMA) both during motor and internal timing tasks. Opposing findings, however, have been shown for the modulation of beta oscillations in the SMA. While movement suppresses beta oscillations in the SMA, motor and non-motor tasks that rely on internal timing increase the amplitude of beta oscillations in the SMA. These independent observations suggest that the relationship between beta oscillations and BOLD activation is more complex than previously thought. Here we set out to investigate this rapport by examining beta oscillations in the SMA during movement with varying degrees of internal timing demands. In a simultaneous EEG-fMRI experiment, 20 healthy right-handed subjects performed an auditory-paced finger-tapping task. Internal timing was operationalized by including conditions with taps on every fourth auditory beat, which necessitates generation of a slow internal rhythm, while tapping to every auditory beat reflected simple auditory-motor synchronization. In the SMA, BOLD activity increased and power in both the low and the high beta band decreased expectedly during each condition compared to baseline. Internal timing was associated with a reduced desynchronization of low beta oscillations compared to conditions without internal timing demands. In parallel with this relative beta power increase, internal timing activated the SMA more strongly in terms of BOLD. This documents a task-dependent non-linear relationship between BOLD and beta-oscillations in the SMA. We discuss different roles of beta synchronization and desynchronization in active processing within the same cortical region.

## Introduction

Regular time intervals reflect fundamental characteristics of rhythmic events that the brain uses to optimize perception and motor behavior. Anticipation of future events after having internalized temporal regularities in the sensory input is called internal timing (Nobre et al., 2007) or predictive timing (Arnal and Giraud, 2012). Both cerebral networks that serve rhythm perception and production as well as neural oscillations that serve this function have been identified. Yet, conflicting reports exist regarding the relationship between neural oscillations and BOLD signal associated with internal timing. In this study, we investigated the relationship between neural beta oscillations and activation as measured by BOLD in the cortical core region of rhythm processing, the supplementary motor area (SMA).

Several empirically grounded models propose the SMA, embedded in a cortical and subcortical network, plays a major role in internal time keeping which is a prerequisite for rhythm processing (Schubotz, 2007; Kotz et al., 2009; Large et al., 2015). Neuroimaging studies documented SMA activation for internal time keeping during rhythm perception (Schubotz et al., 2000; Grahn and Brett, 2007; Grahn and Rowe, 2009) and rhythmic finger tapping (Larsson et al., 1996; Jäncke et al., 2000; Lewis and Miall, 2002; Wiener et al., 2010). Electrophysiological studies focusing on the spectral features of the neural signal of the SMA during rhythm processing identified effects mainly in oscillations in the beta frequency range. In rhythmic finger tapping, rhythmic beta amplitude variations and increased beta coherence between primary motor areas and the SMA have been observed, especially for internally paced tapping (Gerloff et al., 1998; Pollok et al., 2005; Boonstra et al., 2006). Motor beta oscillations may also contribute to auditory rhythm perception even in the absence of overt movement, suggesting an active role in coding temporal predictions (Fujioka et al., 2012, 2015; but see Meijer et al., 2016).

On the other hand, the beta rhythm has been considered an idling rhythm in the motor system because beta oscillations increase in synchrony during rest (Pfurtscheller and Lopes da Silva, 1999). Beta rhythms desynchronize before and during a movement and resynchronize after task completion in sensorimotor cortex (Pfurtscheller, 1981; Salmelin et al., 1995; McFarland et al., 2000; Neuper and Pfurtscheller, 2001). An electrocorticography study documented a similar pattern in the SMA between 18 Hz and 22 Hz (Ohara, 2000). This suggests a negative relationship between the amplitude of beta oscillations and BOLD activation over central regions including the SMA, particularly during motor tasks. Yet, while beta rhythms in sensorimotor cortices are suppressed during movement preparation and execution, beta power increases in motor cortices during anticipation of an upcoming sensory event (Kilavik et al., 2013). Specifically over the SMA, beta power increases have been reported for time estimation in a working memory task between 14 Hz and 30 Hz (Kulashekhar et al., 2016). This beta power increase would argue for a positive relationship between amplitude of beta oscillations and BOLD activation in the SMA.

Together, both beta power decreases and increases have been associated with active processing in the SMA. Task-based EEG-fMRI studies usually reveal relationships between the BOLD signal and EEG power envelope modulations during repeated alternations between a single condition and rest. These studies revealed expectedly a strong negative relationship between beta power and BOLD activation during motor tasks mostly in primary motor cortices but also in the SMA (Formaggio et al., 2008, 2010; Ritter et al., 2009; Yuan et al., 2010; Sclocco et al., 2014). Some studies also report positive correlations between the power of beta oscillations and the BOLD signal; however, these findings are far smaller in size and less consistent with regard to the effect location (Ritter et al., 2009; Scheeringa et al., 2009). The relationship between beta oscillations and BOLD activity in the SMA during internal rhythm generation is unclear.

This study addresses these prima vista opposing findings by simultaneously acquiring EEG and fMRI data during a rhythmic finger tapping task. Here we study the relationship between beta power and the BOLD signal beyond movement-related beta desynchronization. Specifically, we investigated internal timing-related effects in the beta band during several tapping conditions with varying demands on internal rhythm generation. In an auditory-paced finger tapping task, participants were either asked to tap on every auditory stimulus (fast tapping rate, F) or on only every fourth identical auditory stimulus (slow tapping rate, S). While the tapping rate changed for slow and fast conditions, the auditory stimulus did not change across conditions. We hypothesized contrasting the internal generation of a slow rhythm during slow tapping against simple auditory-motor synchronization during fast tapping revealed effects associated with internal timing, because the slow tapping rate was generated internally in the presence of a constant stream of auditory stimuli.

To test for effector effects on SMA activity, tapping was either performed with the left and the right hand. While effects of hand have already been shown for the primary motor areas in electrophysiological and fMRI studies (Jäncke et al., 2000; McFarland et al., 2000; Boonstra et al., 2006; Hayashi et al., 2008), effects of hand on beta power in the SMA, however, remain unclear. One could envisage stronger engagement of the SMA during left compared to right hand tapping based on the left-dominant control of both left and right hand unimanual actions with the concomitant increase in interhemispheric information transfer (Schluter et al., 2001; Rushworth et al., 2003).

The study was conducted only in right-handed participants since we were not interested in the effect of left-handedness on brain activity. Also, this study focuses only on beta band power fluctuations because this frequency range has previously been related with internal timing effects (Gerloff et al., 1998; Pollok et al., 2005; Boonstra et al., 2006; Fujioka et al., 2012, 2015) even though there is evidence that also alpha and gamma oscillations contribute to explaining variance in the BOLD signal (Scheeringa et al., 2011).

Beta oscillations have been subdivided in relative lower and relative higher frequency ranges. While exact frequency boundaries differ from study to study, low beta oscillations have been associated with long-distance multimodal integration and top-down processing (von Stein et al., 1999; Kopell et al., 2000; Lee et al., 2013; Bressler and Richter, 2015). High beta oscillations have been observed during movement preparation and sustained movements (Farmer, 1998; Roopun et al., 2006). Yet, beta oscillations over the SMA have been reported in various frequency ranges (Kaiser et al., 2000; Ohara, 2000; Neuper and Pfurtscheller, 2001; Pfurtscheller et al., 2003; Fujioka et al., 2012, 2015). We thus determined low and high beta frequency ranges in our sample and thus analyzed frequency bands from 14 Hz to 24 Hz and 25 Hz to 35 Hz separately.

We hypothesized a general beta power decrease across all tapping conditions due to movement generation. Relative increases in beta power in an overall beta-suppressed state, however, should be observed in conditions with higher demands on internal rhythm generation as in slow tapping conditions. At the same time, higher BOLD activity in the SMA should be observed for conditions requiring internal slow rhythm generation. Potentially, the SMA could activate more strongly and beta power could decrease more strongly for left compared to right hand tapping, since left hand tapping requires more interhemispheric information transfer, because unimanual hand control is left-dominant (Schluter et al., 2001; Rushworth et al., 2003).

## Materials and Methods

### Participants

Twenty-five participants (10 males; aged 19–31 years; mean 23.8 years) were included in the EEG-fMRI study. Participants had normal or corrected-to-normal visual acuity, no neurological deficits and were right-handed according to self-reports and their scores on the Edinburgh inventory of manual preference (mean handedness quotient 85.5, Oldfield, 1971). Participants performed a test run before measurement to become familiar with the task. All participants gave their written informed consent prior to the study and were paid for participation. The study was approved by the local ethics committee of the Medical Faculty of Goethe University Frankfurt (GZ12/14) and is in accordance with the Declaration of Helsinki.

### Auditory-Paced Finger Tapping Paradigm

**Figure 1:**
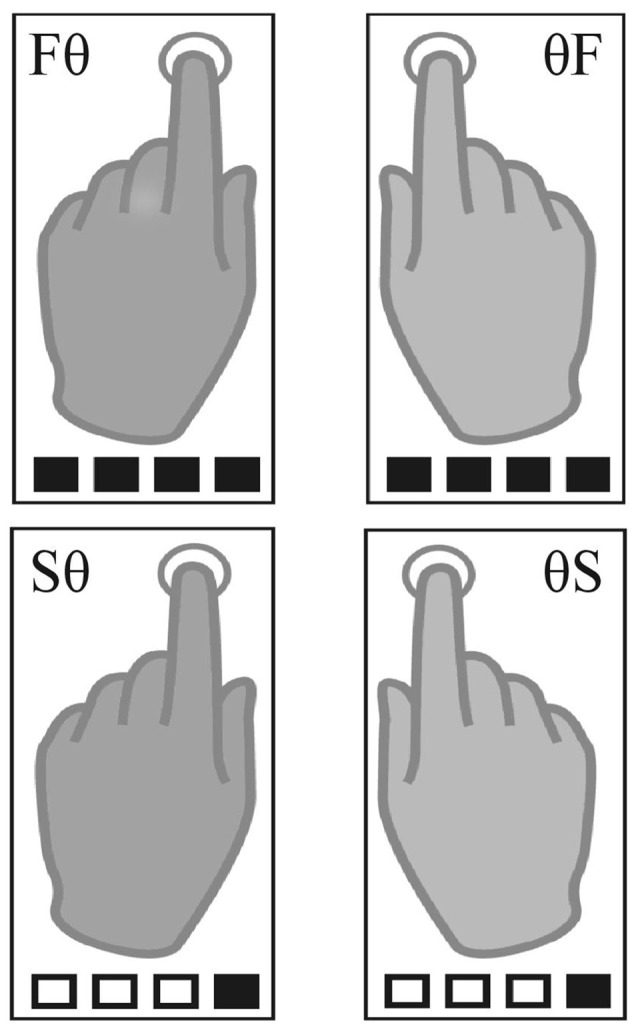
Task conditions. The four unimanual conditions included two left-hand (left fast, Fθ; left slow, Sθ) and two right-hand conditions (right fast, θF; right slow, θS). Squares illustrate the auditory beat; black filled squares indicate tapping.

The paradigm was adapted from Pflug et al. (2017). Using MR-compatible headphones, auditory beats (1.6 kHz, 2 ms duration) were binaurally presented with a constant inter-onset-interval of 400 ms (2.5 Hz, 150 bpm) in all conditions. Participants were asked to tap with their index fingers at a slow or a fast rate synchronized to un-accentuated auditory beats. The fast tapping rate was defined as tapping to every beat. For slow tapping rates, participants were instructed to iteratively count four beats internally and tap only on only every fourth beat. While fast tapping represented simple auditory-motor synchronization, slow tapping required internal generation of a slow rhythm. The auditory stimulus, however, was identical for all conditions. We report here the results of four unimanual conditions that differed in tapping rate/hand mappings (Figure 1). Participants used one hand for tapping the slow or the fast rate while the other hand was not moving (left slow, Sθ; right slow, θS; left fast, Fθ; right fast θF). Please note that there were four additional bimanual conditions during which participants were instructed to tap with both hands the same rate (both fast or both slow) or tapped both rates in parallel (right hand fast and left hand slow rate and vice versa). To reduce complexity, we report here only the unimanual conditions.

Tapping was performed in pseudo-randomized blocks. Tapping blocks lasted 15 s during which 36 auditory beats were presented. This resulted in 36 finger taps in fast conditions and nine finger taps in slow conditions. Before each block, a visual cue indicated the upcoming condition (jittered between 4.6 s and 5.6 s). For each hand, a downward arrow indicated slow tapping, an upward arrow fast tapping, and a dot a stationary hand (left arrow for the left, right arrow for the right hand e.g., ↑ • = Fθ). Four auditory beats of higher pitch primed the fast tapping rate and tapping onset. An inter-block-interval, during which a fixation-cross was presented, was jittered between 5.5 s and 8 s. All eight conditions were presented six times in randomized order, resulting in a total of 48 blocks lasting 22 min.

### Experimental Setup

A projector was used to display the visual stimuli on a screen that participants viewed via an MR compatible mirror and auditory beats were presented using MR-compatible headphones (MRConfon, Magdeburg, Germany). The auditory beats and the visual instructions were presented with Presentation software (Neurobehavioral Systems, Albany, CA, USA, RRID: SCR_002521). Participants were asked to restrict their gaze to the center of the screen during the task. Two pneumatic Biopac pressure sensors (module of model MP150, BIOPAC Systems Inc., Goleta, CA, USA, RRID: SCR_014279) were used to record participants’ tapping pressure. These were attached to the pads of participants’ index fingers. Participants were lying in a supine position in the MR bore and were asked to tap with their index fingers on their ipsilateral thigh. The pressure sensitivity of the sensors was 0.01 cm H_2_O with a sampling rate of 1 kHz. Pressure data were inspected for tapping errors. They were below 2% in each participant; thus, no participant was excluded from further analyses.

### EEG Data Recording and Preprocessing

During scanning, EEG was recorded using a BrainAmp MR EEG amplifier (Brainproducts, Gilching, Germany) and a BrainCap electrode cap (EASYCAP, Herrsching, Germany) with 30 EEG and 2 EOG channels. Ag/AgCl EEG ring electrodes were positioned according to an extended 10/20 system with a reference electrode placed between Fz and Cz as used in Viola et al. (2009). The impedance of all EEG electrodes was kept below 10 kΩ after preparation. ECG, for cardioballistic artifact correction, and surface EMG, from both extensor digitorum communis muscles, was recorded using an additional BrainAmp ExG MR amplifier with the corresponding EMG connecting device (ExG Aux box). Raw EEG data was sampled at 5 kHz with a range of ±16.384 mV, a low-pass filter of 250 Hz, and a high-pass filter of 0.1 Hz using the Brain Vision Recorder software. The EEG data recording was synchronized via a SyncBox to the MR scanner clock to improve artifact correction. The entire equipment was MRI compatible and met all security standards (Brain Products EEG-fMRI Hardware, RRID: SCR_009443).

EEG off-line artifact correction was performed in Brain Vision Analyzer software (Version 2.1, Brainproducts) according to standard preprocessing procedures (for details see Allen et al., 1998, 2000; Jahnke et al., 2012). In brief, gradient artifacts were automatically detected and subsequently subtracted from the data. After down-sampling the data to 250 Hz, R-peaks in the ECG channel were automatically marked and used for correction of cardioballistic artifacts. Before performing an independent component analysis (ICA) to remove additional cardioballistic artifacts, horizontal eye movements and eye blinks, the data were low-pass filtered at 48 Hz and visually inspected for artifacts and manually marked. Marked artifacts were automatically excluded from the subsequent ICA decomposition. A classical sphering approach within an infomax ICA with a convergence bound of 1 × 10^−1^ and a maximum of 512 steps was applied for matrix decomposition. Finally, all channels were referenced to the average of all EEG channels. For each participant, the length of all visually marked artifacts was summed up and set in relation to the total block duration. The entire data set was discarded when more than 10% of the data were affected by artifacts (Laufs et al., 2003). By applying these rules, 5 of the 25 participants were excluded from further analyses.

### EEG Power Analysis

To investigate effects of the tapping task on the EEG power spectrum, power spectral density was calculated from the mean signal of EEG electrodes that are sensitive to signal of the SMA (F3, F4, Fz, FC3 and FC4). These electrodes were defined using an independent EEG/MEG measurement in 17 participants from which eight participants also participated in the current EEG/fMRI study. MEG was recorded using a whole-head system (Omega 2005, VSM MedTech) with 275 channels at a sampling rate of 1200 Hz. Simultaneous EEG was recorded with a custom-made cap equipped with 64 MEG-compatible AG/AgCL electrodes (EASYCAP, Herrsching, Germany). Participants performed the identical finger tapping paradigm during parallel EEG/MEG recording which allows for proper source analyses. MEG and EEG data were filtered off-line with fourth-order Butterworth 300 Hz low-pass and 2 Hz high-pass filters. Line noise at 50 Hz was bandpass-filtered. Recorded data were down-sampled to 1000 Hz. Blocks containing muscle and SQUID artifacts were removed using an automatic artifact rejection algorithm (Oostenveld et al., 2011). Blocks with a head movement exceeding 5 mm were also discarded from further analysis. An ICA was used to identify and reject components of heart muscle and blinks in MEG and EEG data separately. EEG channels were visually inspected and channels containing noise were discarded before EEG signals were re-referenced to a common average. Only valid blocks of both modalities were used for further analysis, which resulted in 8–15 blocks per condition and subject (mean 12 blocks) for analysis.

**Figure 2:**
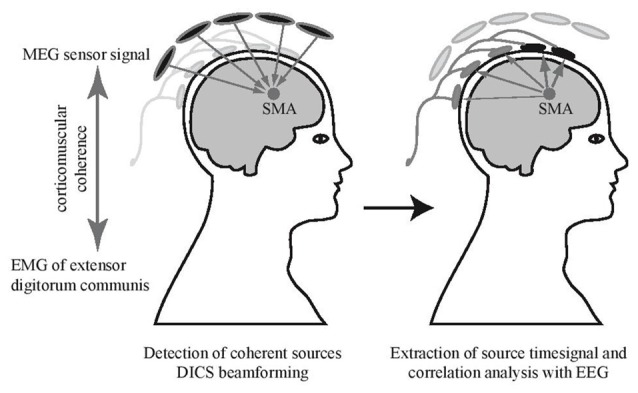
Electrode selection. Supplementary motor area (SMA) source signals obtained from MEG beamforming were used for a correlation analysis with simultaneously recorded EEG. Channels with significant correlation were selected (marked in black) and corresponding EEG electrodes in the EEG-fMRI experiment were used for further analyses.

For source identification, the mean activity of the left and the right extensor digitorum communis muscle during the bimanual fast tapping condition was used as an external reference signal to detect coherent MEG sources in the brain (see Figure 2; Gross et al., 2001; Pollok et al., 2005; Muthuraman et al., 2014). A DICS beamformer (Gross et al., 2002) approach was used to identify sources with highest coherence with the EMG data. The source time series in the SMA were extracted for every condition using the LCMV beamformer method (Van Veen et al., 1997) in a frequency band from 2 Hz to 300 Hz. To detect EEG channels sensitive to the SMA source activity, the correlation between MEG source signal and EEG electrodes was calculated (Figure 2). To allow for statistical comparisons, surrogate datasets were created with block sequence being randomized for EEG signals but kept constant for MEG source signals. Surrogate values and real correlation values were compared using a paired sample *t*-test, significance was assumed at *p* < 0.05. This revealed that the EEG electrodes F3, F4, Fz, FC3 and FC4 correlated with activity in the SMA. To assess that signal from close-by cortical sources did not influence signal in the selected electrodes relevantly, we correlated also time courses of both left and right dorsal premotor cortices with the EEG signal. Time courses in these sources did not correlate significantly with activity in the set of SMA-sensitive electrodes (both *p* > 0.05). More detailed results of the MEG data will be presented elsewhere.

Thus, EEG power in a frequency window from 2 Hz to 40 Hz was analyzed in F3, F4, Fz, FC3 and FC4 separately for the conditions and baseline using a multitaper frequency transformation (hanning window). Due to residuals of scanner artifacts in high-frequency ranges (as described in von Wegner et al., 2016) analyses were restricted to a maximum frequency of 40 Hz. The baseline was defined as the inter-block-interval during which participants were neither tapping, nor instructed for an upcoming tapping block. Mean power across electrodes was plotted over frequencies, separately for each condition and baseline. Peaks in the theta and alpha range included harmonics of the auditory stimulation rate at 2.5 Hz and were thus not further analyzed. Power spectra revealed expectedly two beta components: low beta peaked between 19 Hz and 20 Hz and high beta between 29 Hz and 30 Hz with a trough at 25 Hz between them. These peaks did not correspond to harmonics of 2.5 Hz oscillations or of multiples of the scanning sequence’s repetition time of 2.08 s. We thus further studied power in the low and high beta band separately in frequency windows between 14 Hz and 24 Hz and 25 Hz and 35 Hz.

First, all tapping conditions were compared with fixation baseline using paired *t*-tests (*p* < 0.05, Bonferroni corrected for multiple comparisons which results in a significance level of *p* < 0.0125) separately for mean power differences in low (14–24 Hz) respective high (25–35 Hz) beta. Second, condition differences were investigated using a 2 × 2 ANOVA with timing (internal slow rhythm generation, auditory-motor synchronization) and hand (left, right) as factors. *Post hoc t*-tests were additionally performed for significant main effects (*p* < 0.05, Bonferroni corrected for multiple comparisons). All analyses were performed in SPSS for low and high beta separately (IBM SPSS Statistics, RRID: SCR_002865).

### fMRI Recording and Preprocessing

Image acquisition was performed on a Siemens Trio 3 Tesla magnetic resonance system (Siemens MAGNETOM Vision, Erlangen, Germany) equipped with a circular polarized Send/ Receive head coil with an integrated preamplifier. Functional images were obtained with a gradient-echo T2*-weighted transverse echo-planar imaging sequence (614 volumes; repetition time (TR) = 2.08 s; echo time (TE) = 29 ms; flip angle = 90°; 32 axial slices in descending order; 3 mm × 3 mm × 3 mm isotropic voxel size). Additionally, high-resolution T1-weighted anatomical scans (TR = 2.25 s; TE = 3.83 ms; flip angle = 9°; 176 slices per slab; 1 mm^3^ isotropic size) were obtained. To reduce head motion, a vacuum cushion was used (Vac Fix System, Avondale, AZ, USA).

Image processing and data analyses were performed in SPM12 (Welcome Trust Centre for Neuroimaging, London, UK; RRID: SCR_007037). After eliminating the first four volumes in each participant due to field inhomogeneity of the scanner in the beginning of each run, standard preprocessing was performed (realignment, co-registration of anatomical T1-images to the mean functional image with subsequent segmentation using Tissue Probability Maps, normalization to the Montreal Neurological Institute (MNI) standard brain template, and smoothing with an 8 mm full-width at half-maximum Gaussian kernel). The preprocessed images were analyzed within the framework of general linear models (GLM) for time-series data (Worsley and Friston, 1995).

### fMRI Region of Interest Analysis in the SMA

To investigate condition-related BOLD effects associated with internal timing in the SMA, a fMRI region of interest analysis was performed. On the single-subject level, four condition-specific regressors of interest (for Sθ, θS, Fθ, θF) in addition to four regressors of no interest (bimanual conditions) were modeled by convoluting the onsets and durations of conditions (modeled by boxcar functions) with the canonical hemodynamic response function to obtain predicted BOLD responses. Additional nine regressors of no interest were capturing the variance associated with the instructions for each conditions and an additional tap participants usually made after the last metronome click in fast conditions. Six non-convolved regressors were modeling head-motion-related effects.

For group-level analyses, the four regressors of interest, modeling condition-specific tapping effects, were included in a 2 × 2 ANOVA, similar to the aforementioned EEG analysis. The SMA coordinate (0/0/70, x/y/z, MNI space) reflected the MNI coordinate of the MEG source (see above). Because the coordinate falls in the inter-hemispheric fissure, two cubic ROIs were used to extract fMRI beta values from the left and right SMA, separately (left SMA: -10–0/-10–0/60–70, min.–max. x/min.–max. y/min.–max. z, MNI space and right SMA: 0–10/-10–0/60–70, min.–max. x/min.–max. y/min.–max. z, MNI space). For each of the four conditions of interest, average beta values for the two ROIs were extracted from all 20 subjects with their respective standard error of the mean (MarsBaR region of interest toolbox for SPM, RRID: SCR_009605). Analyses were performed for the left and right SMA separately in SPSS (*p* < 0.05, IBM SPSS Statistics, RRID: SCR_002865).

## Results

### EEG Power Analysis

**Figure 3:**
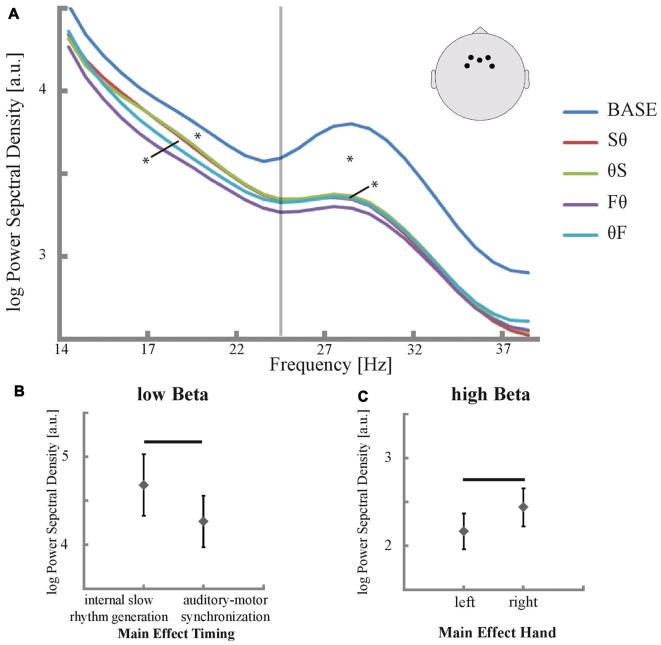
Power in EEG electrodes that are sensitive to activity in the SMA and their significant main effects. **(A)** Mean power spectral density was plotted between 14 Hz and 40 Hz for all conditions and baseline. Significant differences in low and high beta power of conditions compared to baseline are marked with an asterisk (*p* < 0.05, corrected for multiple comparisons) as well as significant main effects of time in low beta power and of hand in high beta power (*p* < 0.05). The positions of the selected electrodes F3, F4, Fz, FC3 and FC4 are illustrated. The vertical line indicates the trough in the power spectrum between low and high beta at 25 Hz.** (B,C)** Main effect of timing respective main effect of hand for low respective high beta power (*p* < 0.05). Significant differences between internal slow rhythm generation (Sθ and θS) and auditory motor synchronization (Fθ and θF) as well as differences between left and right hand are marked with a black bar (*p* < 0.05, corrected for multiple comparisons).

The condition-specific power spectral density between 15 Hz and 40 Hz in electrodes over the SMA is depicted in Figure 3. For low and high beta, all tapping conditions showed a reduction in power compared to baseline (all *p* < 0.007, corrected for multiple comparisons, Figure 3A). More importantly, the ANOVA revealed a main effect of timing in the low beta band (*p* = 0.023, *F* = 6.163, Figures 3A,B) while for the high beta band no effect of timing was found (*p* = 0.343, *F* = 0.946). *Post hoc t*-tests in the low beta band revealed higher low beta power in slow than in fast tapping conditions (*p* = 0.031, corrected for multiple comparisons, Figure 3B). Conversely, a main effect of hand was found for the high beta band (*p* = 0.034, *F* = 5.228, Figures 3A,C) but not in the low beta band (*p* = 0.156, *F* = 2.184). In the high beta band, *post hoc t*-tests revealed stronger power decreases for the left than for the right hand (*p* = 0.031, corrected for multiple comparisons, Figure 3C). No interactions between timing and hand were found—neither in the low nor in the high beta band (all *p* > 0.05). Yet, the hand effect resulted primarily from the F0 condition, which showed the strongest high beta power decrease (Figure 3A).

### fMRI Activation

**Figure 4:**
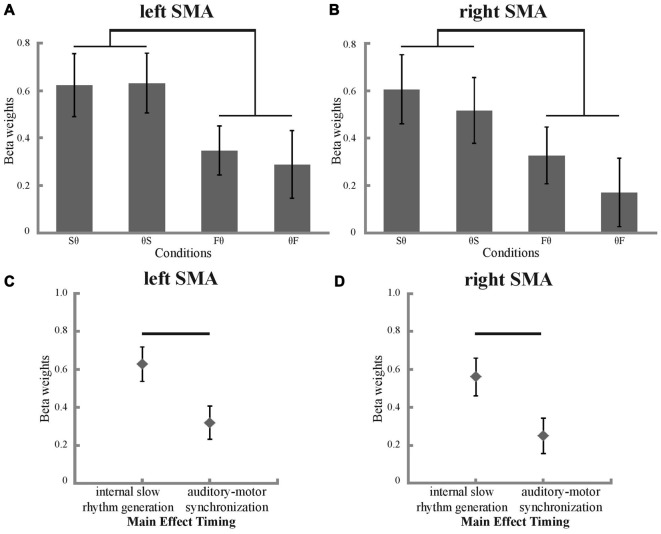
Beta weights and their significant main effect of timing in the SMA. Condition-specific beta weights are plotted separately for the left **(A)** and right **(B)** SMA. Significant differences between internal slow rhythm generation (Sθ and θS) and auditory motor synchronization (Fθ and θF) are marked with a black bracket (*p* < 0.05, corrected for multiple comparisons). Main effect of timing in the left **(C)** and right **(D)** SMA (*p* < 0.05). Significant differences between internal slow rhythm generation (Sθ and θS) and auditory motor synchronization (Fθ and θF) are marked with a black bar (*p* < 0.05, corrected for multiple comparisons).

To investigate BOLD signal condition differences in the SMA, ROI analyses were performed. As expected, all conditions showed more activation compared to fixation baseline (Figures 4A,B). A main effect of timing was found in the ANOVA, both for the left and right SMA (left SMA: *p* = 0.003, *F* = 11.188; right SMA: *p* = 0.004, *F* = 10.917; Figures 4C,D). *Post hoc t*-tests revealed stronger activation for internal slow rhythm generation than for auditory-motor synchronization in both the left and right SMA (left SMA: *p* = 0.003, corrected for multiple comparisons; right SMA: *p* = 0.004, corrected for multiple comparisons, Figures 4A,B). Neither the left nor the right SMA showed a significant main effect of hand or significant interactions between hand and timing (all *p* > 0.05).

## Discussion

Our study revealed four key findings in the SMA. First, the comparisons against baseline revealed the well-known condition-independent task-related desynchronization of low and high beta oscillations together with a concomitant BOLD activation. Second, within the overall suppressed beta state of the SMA during tapping, generation of an internal rhythm relatively increased beta amplitude in parallel to a concordant increase in BOLD activation. Third, and as a consequence of finding one and two, no general linear relationship between the EEG signal in the beta band and the BOLD signal was found. Fourth, tapping with the left compared to right hand decreased beta power in the SMA, particularly for fast tapping, while this effect was not observed in BOLD.

When investigating SMA activity-sensitive EEG electrodes for condition effects in relation to baseline, beta desynchronization was found for all conditions in the low (14–24 Hz) and high beta band (25–35 Hz). Since these effects were condition-independent they likely relate to a general task-related activation of the SMA. Indeed, BOLD analyses documented such activation that has previously been related to movement effects in fMRI finger tapping studies (Rao et al., 1993; Jäncke et al., 2000; Meister et al., 2005; Witt et al., 2008). The negative relationship between beta power and BOLD signal increase in the SMA and other motor-related cortices is known for motor tasks, but has also been documented in the inferior frontal gyrus and parietal cortices (Formaggio et al., 2008, 2010; Ritter et al., 2009; Yuan et al., 2010). It is thus likely that movement planning, anticipation, or execution decreases beta power and increases BOLD activity in the SMA.

The second key finding breaks the inverse relationship between beta power and BOLD activation in the SMA. Internal timing increased, both, beta power and fMRI activation of the SMA in comparison to conditions with low internal timing demands. The increase of activation is in line with findings in which SMA activation has been associated with internal time keeping (for review see Grahn and Rowe, 2009; Wiener et al., 2010). EEG effects associated with internal timing were only observed in the low beta band in our study. This specifies previous proposals on the contribution of beta oscillations to predictive timing (Schubotz, 2007; Arnal and Giraud, 2012; Bartolo et al., 2014; Bartolo and Merchant, 2015; Kulashekhar et al., 2016; Morillon et al., 2016). Beta oscillations in sensorimotor cortex increase in power in anticipation of future events whenever temporal predictions are possible (Kilavik et al., 2013). This suggests that beta oscillations may serve as a timing mechanism not only in rhythmic conditions but rather serve as an internal clock to predict timing of future events even when they do not occur rhythmically. Rhythmic signals from the motor cortex could reset timers in sensory cortices to improve temporal predictions of future sensory events (Fujioka et al., 2012, 2015; Morillon et al., 2014; Morillon and Schroeder, 2015). Information passing between brain regions in large scale neural networks including motor cortices cycles in beta frequencies, suggesting that the motor system receives and/or transmits information to other brain regions in beta-long segments (Picazio et al., 2014). Indeed, motor cortices form large scale neural networks with distant cortical regions by means of beta synchronization (Roelfsema et al., 1997; Gehrig et al., 2012; Bressler and Richter, 2015).

The opposing observation of parallel movement-related beta desynchronization and internal timing-related beta synchronization in the same activated cortical region could be explained if both power modulations (in comparison to baseline and in comparison to the movement-related suppression without internal timing demands) were interpreted as active processes. A recent neurodynamic model focusing on cortico-hippocampal interactions during memory encoding proposes an innovative perspective on the active role of beta desynchronization even in non-motor cortices (Hanslmayr et al., 2016). Neocortical alpha and beta desynchronization may interact with theta-gamma synchronization in the hippocampus, which results in long-term potentiation and memory formation. This model proposes an increase in information processing, as measured by neuronal firing rates, with neocortical beta power decrease. The baseline in our experiment, which required no movement, could be regarded as a perfectly predictable condition during which beta oscillations in motor cortices idle because no computations are required. Activation of a given cortical region could potentially result in beta synchronization in local neural ensembles that are out of phase of neighboring patches within a given cortical region. This would in turn appear as a strong desynchronization when measuring the entire cortical region on the mesoscopic level. If indeed beta oscillations in the motor-related cortices carry time information then this information would likely be used by many neural ensembles within a given region and would additionally be disseminated to other cortical regions. This would result in the here observed beta power increases for conditions requiring additional internal timing that were embedded in an overall movement-related beta suppression. Interestingly, the timing effects were found consistently only in the low beta band, while both beta bands were suppressed in every condition compared to baseline. This suggests that particularly the low beta band carries temporal information and could be used for predictive timing (Kopell et al., 2000; Lee et al., 2013).

Earlier studies associated the simultaneous occurrence of cortical synchronization and desynchronization in alpha and low beta bands with processing in thalamo-cortical networks (Pfurtscheller and Lopes da Silva, 1999; Suffczynski et al., 1999). Subcortical brain regions contribute substantially to rhythm processing, as evidenced by lesion studies or observations in Parkinson’s disease patients and interactions between the SMA, the basal ganglia, and the cerebellum are thought to underlie efficient control of rhythmic movements (Riecker et al., 2005; Schwartze et al., 2012, 2016).

The diminished beta band desynchronization for slow compared to fast finger tapping could potentially also be interpreted as a consequence of different tapping rates. Yet, if reduced motor activity in slow compared to fast tapping would relate to the observed beta power increase, consequences of rate effects would also apply to the fMRI results. Higher tapping rates are linearly related with BOLD increases, however, in primary motor cortices (Hayashi et al., 2008). The current fMRI BOLD analysis revealed the opposite effect in the SMA: less instead of more activation for fast conditions. This excludes tapping-rate effects.

While effects of internal timing were observed in the low beta band, effects of hand were limited to the high beta band. This is not only in accordance to previous findings, which associate finger movement to oscillations in the high beta band in the SMA (McFarland et al., 2000; Pfurtscheller et al., 2003), but also extends these findings by showing stronger high-beta desynchronization for the non-dominant left hand in right-handed participants. This observation could be explained by the need for additional interhemispheric information transfer and thus increased processing demands in the SMA in left hand tapping, because unimanual hand motor control seems to be left-dominant (Schluter et al., 2001; Rushworth et al., 2003). The non-dominant left hand is less frequently used for finger tapping. Consequently, left hand tapping could be regarded as more difficult. Indeed, previous electrophysiological findings in tapping and working memory tasks showed stronger beta desynchronization for increasing task difficulty (Mayville et al., 2001; Lundqvist et al., 2011). Of note, the hand effect was only observed in the high beta band and not in BOLD data, which again suggests that not all beta effects translate equally into changes in BOLD activation.

Our results indicate that beta suppression cannot be simply equated with increases in BOLD signal. While the relationship between beta power and BOLD activation may differ from brain region to brain region (Kujala et al., 2014), we show here that even within a single cortical region, contextual task effects change the correlation between beta power and BOLD activity. Since effects of movement-related beta desynchronization and of internal timing-related beta synchronization occurred in parallel in our experiment, both effects could influence the relationship between beta oscillations and BOLD at the same time. This documents non-linear relationships between the BOLD signal and beta oscillations within the SMA for a timing-related finger-tapping task and questions over-simplified associations between neural oscillations and the BOLD signal.

### Limitations

Internal timing-related effects have mostly been associated with oscillations in the beta band (Gerloff et al., 1998; Pollok et al., 2005; Boonstra et al., 2006; Fujioka et al., 2012, 2015); thus, this study addresses the relationship between beta band power fluctuations and BOLD. However, investigations in other frequency bands as alpha or gamma would also be of interest since alpha and gamma oscillations also contribute to explaining BOLD variance (Scheeringa et al., 2011). It would also be of interest to investigate movements with a wider range of internal timing demands. A parametric design using lower and higher-order tapping rates, representing different levels of internal-timing demands, could be used to further quantify the observed timing effect.

Our study design included eight conditions and was thus not ideally designed for classical EEG-fMRI analyses of correlations between the BOLD signal and EEG power fluctuations over time, especially with respect to analyses of correlations between the beta power envelope beyond task-related desynchronizations with BOLD activity. Such an analysis requires larger time windows for correlation compared to the block length used in this study. With the knowledge of internal timing-related effects for unimanual conditions, a reduced task design with only unimanual conditions and longer tapping blocks could be more appropriate to capture such effects using standard analyses.

The EEG electrode selection was based on significant correlation with MEG source signal in the SMA. We are thus confident that the signal reflects activity in the SMA. Yet, we cannot exclude that these electrodes also picked up signal from neighboring cortices like slightly more lateral aspects of the dorsal premotor cortex. We believe their contribution is marginal since time courses in the left and right dorsal premotor cortices did not correlate with the set of EEG electrodes reflecting activity in the SMA.

## Conclusion

In conclusion, our findings suggest internal timing-related low beta synchronization occurs in the context of movement-related beta desynchronization in the SMA. The non-linear relationship between beta power effects and BOLD activation is suggestive of active contributions of both regional beta desynchronization and subregional beta synchronization to active processing.

## Author Contributions

This article was written by FG, AP, HL and CAK. FG recorded, preprocessed and analyzed the EEG-fMRI data with help of CAK. AP recorded, preprocessed and analyzed the EEG-MEG data and programmed scripts. HL provided the EEG-fMRI equipment and gave methodological support for EEG-fMRI acquisition and subsequent analysis.

## Conflict of Interest Statement

The authors declare that the research was conducted in the absence of any commercial or financial relationships that could be construed as a potential conflict of interest.
